# p.(Asp47Asn) and p.(Thr62Met): non deleterious LDL receptor missense variants functionally characterized *in vitro*

**DOI:** 10.1038/s41598-018-34715-x

**Published:** 2018-11-09

**Authors:** A. Benito-Vicente, H. Siddiqi, K. B. Uribe, S. Jebari, U. Galicia-Garcia, A. Larrea-Sebal, M. Stef, H. Ostolaza, L. Palacios, C. Martin

**Affiliations:** 1 Instituto Biofisika (UPV/EHU, CSIC) and Departamento de Bioquímica, Universidad del País Vasco, Apdo. 644, 48080 Bilbao, Spain; 2 Progenika Biopharma, a Grifols Company, Derio, Spain

## Abstract

Familial Hypercholesterolemia (FH) is a common genetic disorder caused most often by mutations in the Low Density Lipoprotein Receptor gene (*LDLr*) leading to high blood cholesterol levels, and ultimately to development of premature coronary heart disease. Genetic analysis and subsequent cascade screening in relatives allow diagnosis of FH at early stage, especially relevant to diagnose children. So far, more than 2300 LDLr variants have been described but only a minority of them have been functionally analysed to evaluate their pathogenicity in FH. Thus, identifying pathogenic mutations in LDLr is a long-standing challenge in the field. In this study, we investigated *in vitro* the activity p.(Asp47Asn) and p.(Thr62Met) *LDLr* variants, both in the LR1 region. We used CHO-*ldlA7* transfected cells with plasmids carrying p.(Asp47Asn) or p.(Thr62Met) *LDLr* variants to analyse LDLr expression by FACS and immunoblotting, LDL binding and uptake was determined by FACS and analysis of mutation effects was assessed *in silico*. The *in vitro* activity assessment of p.(Asp47Asn) and p.(Thr62Met) *LDLr* variants shows a fully functional LDL binding and uptake activities. Therefore indicating that the three of them are non-pathogenic LDLr variants. These findings also emphasize the importance of *in vitro* functional LDLr activity studies to optimize the genetic diagnosis of FH avoiding the report of non-pathogenic variants and possible misdiagnose in relatives if cascade screening is carried out.

## Introduction

Familial Hypercholesterolemia (FH) (OMIM 143890) is a common genetic disease caused most often by mutations in the Low Density Lipoprotein Receptor gene (*LDLr*; MIM# 606945). These heterogeneous LDLr variants impair the function of the receptor pathway, leading to high blood cholesterol levels, xanthomas, cholesterol accumulation in peripheral tissues and development of atherosclerosis at an early age, ultimately leading to development of premature coronary heart disease (CHD)^1^. With a heterozygous prevalence as high as 1 in 200 in some populations^2^, FH constitutes one of the most common genetic disorders leading to an increased cardiovascular risk^3^. In recent years, the use of new sequencing technologies such as next-generation sequencing and target exome sequencing has enabled the identification of an increased number of new LDLr variants^4–6^. So far, more than 2300 LDLr variants related to FH have been described (ClinVar database). However, only a minority of these variants have been functionally analysed to evaluate their pathogenicity. An improvement of diagnosis and prognosis of FH patients has been highly recommended by World Health Organization (WHO, 1998). The ability to identify FH patients at the earliest opportunity is both economically and socially beneficial with implications for mortality and morbidity^7^. In 2015, the American College of Medical Genetics and Genomics (ACMG) published an algorithm with the goal of facilitating diagnosis when there is a lack of functional evidence for variant pathogenicity^8^. According to the ACMG algorithm, variants can be classified as: (i) pathogenic, (ii) likely pathogenic, (iii) uncertain significance, (iv) likely benign, or (v) benign.

It is important to note that at present, there is insufficient data about most LDLr variants to support a quantitative assignment of variant certainty to any of these five categories given the heterogeneity of the disease. Thus, distinguishing pathogenic mutations in LDLr from non-pathogenic ones is a long-standing challenge in the field^9,10^. To understand the clinical significance of these variants, *in vitro* functional validation must be performed to enable a definite genetic diagnosis of FH. An accurate genetic diagnosis of FH is not only beneficial for index cases but also for their relatives, since cascade screening is usually carried out in FH diagnosis enabling earlier management. Currently, FH is still underdiagnosed despite the trend toward increased genetic diagnosis.

Recently, accurate and accessible methodologies have been developed to assess LDLr activity *in vitro*^9,11–13^. Thus, experimental reproducibility between laboratories all over the world ensures rigorous verification of all functional studies performed for each variant.

In the present study, we aimed to functionally characterize the effects of missense mutations found in FH patients whose effect on LDLr is not established.

## Materials and Methods

### *LDLr* Variant Selection

Two *LDLr variants* - p.(Asp47Asn) and p.(Thr62Met) - were selected to be functionally characterized for three reasons. (1) There was a possibility of an associated LDL binding defect; (2) these variants had been previously documented in FH patients, and (3) the effect of these variants had not been characterized before. Specifically, p.(Asp47Asn) and p.(Thr62Met) were identified through the ClinVar database (https://clinvarminer.genetics.utah.edu) and have been found in 2 and 9 index cases, respectively, by the LIPOchip^®^ platform^14^ and/or by the SEQPRO LIPO RS^®^ platform from Progenika Biopharma (Derio, Spain), both platforms with the CE mark. Description of the studied variants, conservation and *in silico* predictions are shown in Table 1.

**Table 1:** Description of the studied variant, conservation and *in silico* predictions.

Genetic name	HGVS Nomenclature	Conservation nt	Conservation AA	Grantham distance
c.139G>A	p.(Asp47Asn)	1.00	1.00	23
c.185C>T	p.(Thr62Met)	0.973	0.947	81
**Patogenicity Prediction**				
	**Align GVGD**	**SIFT**	**Polyphen-2**	**Mutation Tester 2**
p.Asp47Asn	C0	Deleterious (score 0)	probably damaging (1)	Disease causing (P:1.0)
p.Thr62Met	C0	Deleterious (score 0.01)	probably damaging (1)	Disease causing (P:1.0)

### Construction of p.(Asp47Asn) and p.(Thr62Met) *LDLr* carrying plasmids

Plasmids containing the p.(Asp47Asn) and p.(Thr62Met) *LDLr* variants were generated by Innoprot (Derio, Spain). Briefly, variants were introduced into the human *LDLr* cDNA (NM_000527.4), using the mammalian expression vector pcDNA3 under control of a SV40 promoter by oligonucleotide site-directed mutagenesis using the QuickChange Lightning mutagenesis kit (Agilent) according to the manufacturer’s instructions. The oligonucleotides used to generate the plasmid carrying p.(Asp47Asn) and p.(Thr62Met) *LDLr* variants was synthesized *in vitro* and subcloned using the restriction enzymes SacII and EcoRI. The presence of the desired nucleotide alteration was confirmed by PCR and restriction enzyme digestion of the appropriate fragments. The integrity of the remaining *LDLr* cDNA sequence of the construct was verified by direct sequence analysis.

### *CHO-ldlA7* Cell culture and transfection

CHO-*ldl*A7 cells not expressing *LDLr* kindly provided by M. Krieger (MIT, MA, USA) were maintained in Ham’s F-12 medium containing 10% FBS, 2 mM L-glutamine and antibiotics (100 units/mL penicillin; 100 μg/mL streptomycin). *LDLr* CHO-*ldl*A7 cells grown into 6- or 24-well culture plates at 80% confluence were transfected with Lipofectamine^®^ LTX-Plus^TM^ Reagent (Invitrogen) following manufacturer’s indication. LDLr functionality was assessed 48 h after transfection.

### Immunodetection of LDLr

Cells were lysed using an ice‐cold buffer containing 50 mM Tris–HCl, pH 7.5, 125 mM NaCl, 1% Nonidet P‐40, 5.3 mM NaF, 1.5 mM NaP, 1 mM orthovanadate, 1 mg/ml complete EDTA-free protease-inhibitor cocktail (Roche), and 0.25 mg/ml Pefabloc, 4‐(2‐aminoethyl)‐benzenesulfonyl fluoride hydrochloride (AEBSF; Roche). Cells were rotated at 4 °C for an hour and centrifuged at 12,000 g during 15 minutes to remove insoluble material. Proteins were fractionated by electrophoresis on non-reducing 8.5% SDS-PAGE for semi-quantitative immunoblotting. Following antibodies were added: rabbit polyclonal anti-LDLr antibody (1:500) (Progen Biotechnik GmbH, Heidelberg, Germany), anti-GAPDH antibody (1:1000) (Nordic Biosite, Täby, Sweden) and horseradish peroxidase-conjugated anti-rabbit antibody (GE Healthcare, Little Chalfont, UK). The primary antibodies were incubated overnight at 4 °C while the secondary antibody incubation was performed at room temperature for an hour. Signals were developed using SuperSignal West Dura Extended Substrate (Pierce Biotechnology, Rockford, IL, USA) in a ChemiDoc XRS (Bio-Rad, Hercules, CA, USA). NIH ImageJ software (http://rsbweb.nih.gov/ij/) was used for band intensity quantification, levels of protein of interest were corrected to GAPDH loading control band intensities. Original blot can be found in Supplementary Fig. S1.

### Lipoprotein isolation

Blood plasma used for lipoprotein purification was collected from healthy individuals after 30 min centrifugation, at 12,000 × g at 4 °C. LDL (1.019–1.050 g/mL) was isolated in a sequential ultracentrifugation by adjusting plasma density to 1.21 g/mL by the addition of KBr. Next, a second PBS buffer was added to the top of the solution. Ultracentrifugation was carried out in a TST 41–14 rotor (Kontron, Germany) at 35,400 rpm for 19 h at 4 °C in a Centrikon T-21X0. The white upper band corresponding to VLDL and the intermediate orange band corresponding to LDL were collected and stored at 4 °C. Isolated lipoproteins were used within 2–3 days after purification. This study was approved by the Research Ethics Committee of the University of the Basque Country (Comité de Ética en la investigación y la práctica docente de la Universidad del País Vasco/Euskal Herriko Unibertsitatea; CEIAB/186/2014/MARTÍN PLÁGARO). Methods were carried out according to the approved guidelines. All participants signed the written informed consent. All experiments were carried out according to relevant guidelines and regulations.

### Lipoprotein labelling

Lipoproteins (LDL and VLDL) were fluorescently labelled with fluorescein isothiocyanate (FITC) as described previously^15^. Briefly, LDL and VLDL were loaded in 0.1 M NaHCO_3_ (pH 9.0) pre-equilibrated Sephadex G-25 column and then incubated with 10 µl/mL FITC (2 mg/mL in DMSO) per lipoprotein millilitre (1 mg/mL) at room temperature under constant gentle agitation for 2 h. After incubation, the non-bounded FITC was eliminated by washing the lipoprotein solution in a Sephadex G-25 column previously balanced in PBS EDTA-free buffer. Protein concentration was determined in all fractions using BSA as standard (Pierce BCA protein assay, Pierce).

### Analysis of LDLr expression by

Expression of LDLr at cell membrane was assessed in a FACScalibur using the following antibodies: Mouse anti-human-LDLr (1:100; 2.5 mg/L; Progen Biotechnik GmbH) and Alexa Fluor 488-conjugated goat anti-mouse IgG (1:100; Molecular Probes) as secondary antibody. The inmunostaining was performed as previously described^11^. Briefly, cells were incubated for 1 hour at room temperature with the primary antibody after consecutive fixing and blocking steps. Cells were finally washed 3 times in PBS-1%BSA and incubated for 1 hour at room temperature with the secondary antibody. Each sample was performed in triplicate and data analysis was acquired with 10,000 events.

### Analysis of LDLr activity (lipoprotein binding and uptake) by FACS

Cells were seeded in 24-well plates, at 10^6^ cells/well and transfected as previously described when optimal concentration was reached. 48 hours after transfection, 20 µg/mL FITC-lipoprotein (VLDL or LDL) was added to the cell culture medium and cells were incubated within 4 hours at 37 °C or at 4 °C to determine LDLr activity and its binding to the different lipoproteins respectively. After incubation, cells were rinsed with PBS supplemented with 1% BSA, fixed in 4% paraformaldehyde for 10 minutes and washed again to remove the remaining fixative.

Lipoprotein uptake was determined by adding Trypan blue solution (0.2% final concentration, Sigma-Aldrich, Steinheim, Germany) to the samples. This procedure allows quenching of the extracellular FITC-signal coming from the non-internalized lipoprotein-LDLr complexes. Geometric mean fluorescence intensity of each sample was determined in a FACScalibur Flow cytometer following the manufacturer instructions^13^. Geometric mean fluorescence intensity of 10,000 events was acquired for each sample. Every assay determination was performed at least three independently times.

### Statistical analysis

All measurements were performed at least 3 times, with n = 3 unless otherwise specified, and results represent the mean ± S.D. The differences between p.(Asp47Asn) and p.(Thr62Met) LDLr variants and wt LDLr were tested by a two-tailed Student’s t-test, P-values < 0.05 were considered statistically significant.

## Results

### *In silico* prediction analysis of pathogenicity of LDLr variants

Three different software programs were used to predict pathogenicity of p.(Asp47Asn) and p.(Thr62Met) variants. Table 1 shows the obtained results, p.(Asp47Asn) and p.(Thr62Met) LDLr variants.were predicted as pathogenic by SIFT and Mutation taster software and as probably damaging by Polyphen-2 software. These algorithms use mainly amino acid conservation analysis and as the conservation of the two amino acids is very high, the obtained predictions were the expected ones. To confirm these predictions, *in vitro* functional validation of these LDLr was next assayed.

### p.(Asp47Asn) and p.(Thr62Met) LDLr variant expression

**Figure 1:**
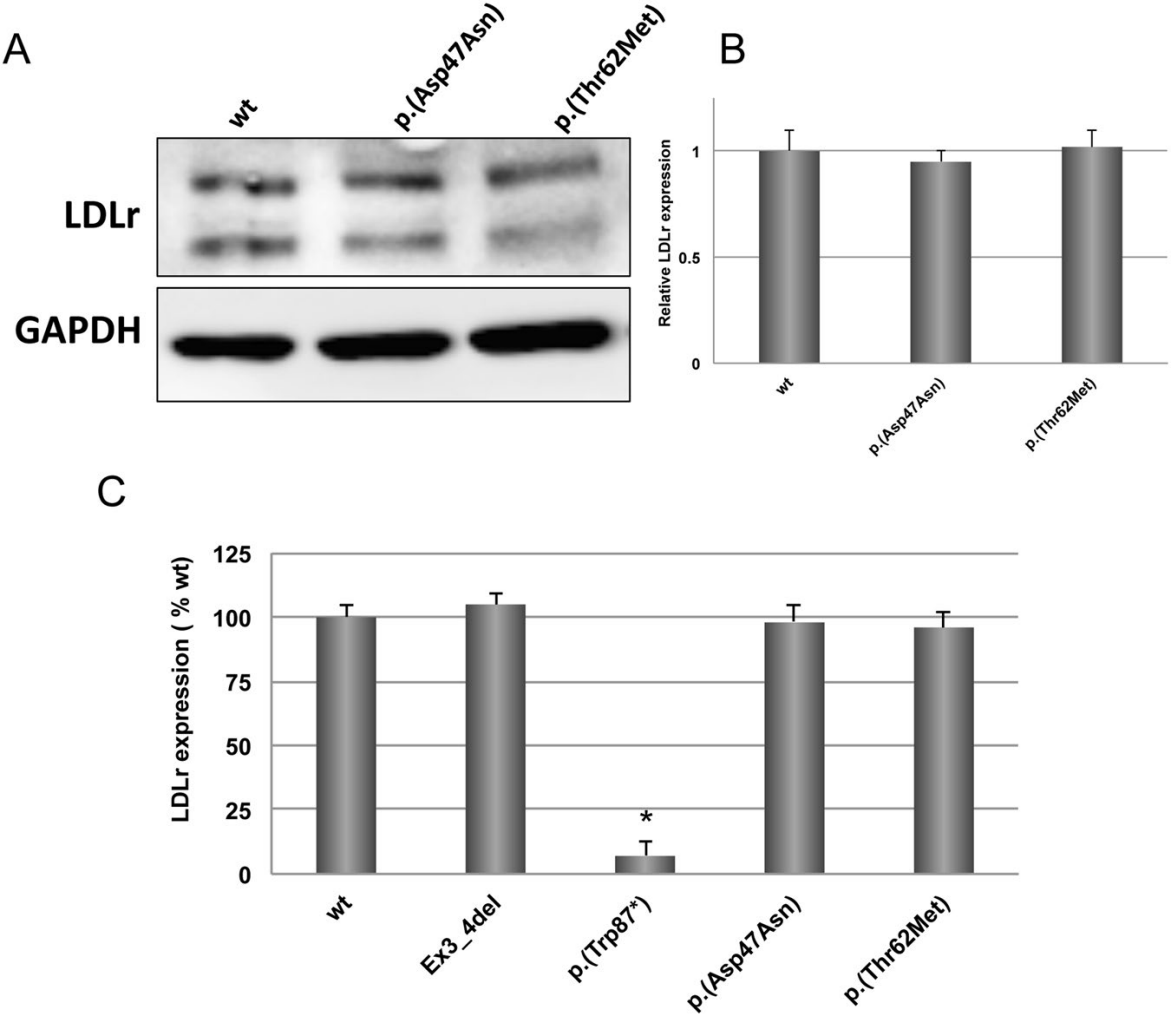
Expression of wt LDLr, p.(Asp47Asn) and p.(Thr62Met) LDLr variants in CHO-*ldlA7* transfected cells. Cells were transfected with the corresponding plasmids, LDLr was overexpressed for 48 h and then cells were lysed and analysed by (**A**) Western blot as described in Materials and Methods section (**B**) relative band intensity of mature LDLr protein expression was calculated as the ratio of 160 kDa LDLr band intensity to that of GAPDH. (**C**) LDLr expression of p.(Asp47Asn) and p.(Thr62Met) LDLr variants determined by FACS, two internal controls were used, p.(Trp87)* (a null allele mutant), and Ex3_4del LDLr variant that produces a defective binding LDLr. A representative experiment from three independently performed assays is shown in A. The differences between p.(Asp47Asn) and p.(Thr62Met) LDLr variants and wt LDLr was determined by a two-tailed Student’s t-test, P-values < 0.05 were considered as statistically significant.in (**B**). The values in (**C**) represent the mean of triplicate determinations (n = 3); error bars represent ± SD. *P < 0.001 compared to the wt using a Student’s t-test.

Expression of p.(Asp47Asn) and p.(Thr62Met) LDLr variant was analysed by Western blot in CHO-*ldl*A7 transfected cells as detailed above. Figure 1A (upper panel) shows that expression of p.(Asp47Asn) and p.(Thr62Met) LDLr variants is similar as wt 48 h post-transfection. Immunoblotting was performed to detect GAPDH protein (Fig. 1A, lower panel) in order to confirm equal loading of cytosolic extracts. Then, quantitative densitometry was performed to determine relative levels of LDLr expression (Fig. 1B). These results were further corroborated by assessing LDLr expression by FACS, as shown in Fig. 1C expression of p.(Asp47Asn) and p.(Thr62Met) LDLr variants, determined by IgG-C7 antibody resulted similar to the wt LDLr.

### p.(Asp47Asn) and p.(Thr62Met) LDLr variant activity

**Figure 2:**
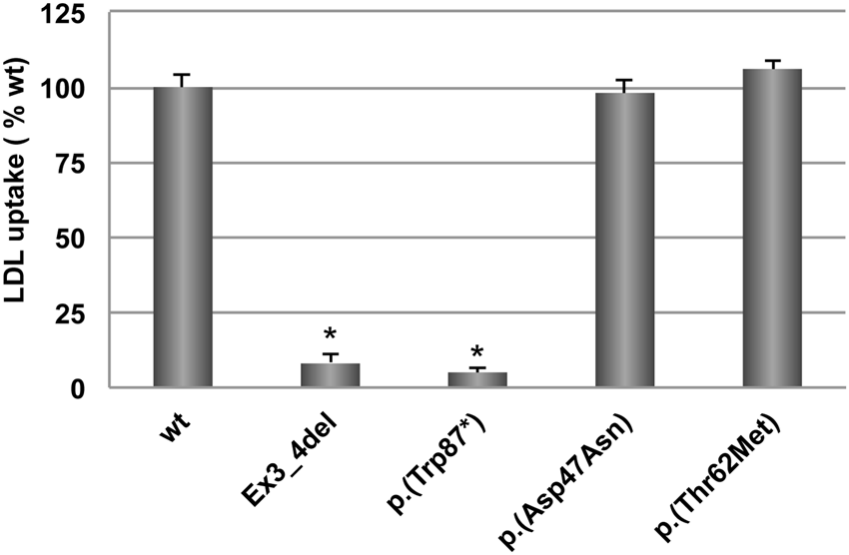
LDLr activity of wt, p.(Asp47Asn) and p.(Thr62Met) LDLr variants. (**A**) LDL-LDLr binding after 4 h incubation at 4 °C; and (**B**) LDL internalisation efficiency after 4 h incubation at 37 °C. Geometric fluorescence intensity of 10,000 events was acquired in a Facscalibur; extent of LDL binding and uptake was calculated as described in Materials and Methods. The values represent the mean of triplicate determinations (n = 3); error bars represent ± SD. *P < 0.001 compared to the wt using a Student’s t-test.

Activity of p.(Asp47Asn) and p.(Thr62Met) LDLr variants was assessed in CHO-*ldl*A7 cells transfected cells. LDL binding and uptake was determined by FACS using p.(Trp87)* (a null allele mutant), and Ex3_4del LDLr variant that produces a defective binding LDLr as internal controls of the assay^16^. As shown in Fig. 2A, LDL-LDLr binding activity of p.(Asp47Asn) and p.(Thr62Met) variants resulted similar than wt LDLr (Fig. 2A). As shown in Fig. 2B, uptake of FITC-labelled LDL in cells expressing p.(Asp47Asn) and p.(Thr62Met) variants was similar to that of wt.

## Discussion

We here report a functional characterization of LDLr activity focused on two LDLr missense variants: p.(Asp47Asn) and p.(Thr62Met), both located at the first cysteine rich domain (LR1) of the ligand binding domain (LBD). The two of them have been previously reported^17–23^. They are included in the ClinVar database by different submitters, 4 and 11, respectively. Both variants are classified as “Uncertain significance” by the majority of them but for both variants there is a submitter who classifies them as “pathogenic”. It is remarkable that all of them are predicted by *in silico* software programs as pathogenic or probably pathogenic, thus ascertaining their activity *in vitro* allows determining their pathogenicity, which is essential for a genetic diagnosis and appropriate clinical counselling.

The LDLr binding domain contains seven cysteine-rich domains (LR1-LR7)^24^, each of which contains about 40 amino acids and, each domain conformation is stabilized by three disulphide bridges^25^. It has been shown that combination of multiple LR’s contributes to lipoprotein recognition and binding, including LR1^26^. As mentioned above, p.(Asp47Asn) and p.(Thr62Met) are located at the LR1 and both are predicted as pathogenic by *in silico* software programs. Despite being previously described, none of these variants had been functionally characterized. In order to enable diagnoses of patients with these LDLr variants, we assessed the activity of p.(Asp47Asn) and p.(Thr62Met) LDLr varians to gain insight into their role in FH development.

A review of the secondary structure adopted by LR1^25,27–29^ shows that Asp47 is involved in a turn formed by residues 46–49 that allow the segment between Cys(IV) and Cys(V) to loop around the side chain of Trp44^29^. According to our data which show no loss of activity of p.(Asp47Asn) LDLr variant, we surmise that the turn is not affected by the amino acid substitution and that LR1 retains correct folding to allow lipoprotein binding.

It is well established that the six cysteines present in each LR interact via Cys(I)-Cys(III), Cys(II)-Cys(V), and Cys(IV)-Cys(VI) disulfide bonds thus allowing the required structure to coordinate one calcium ion. Given the stringency of these Cys-Cys interactions, it is possible that maintaining the loops that allow proper Cys-Cys bonding requires highly restricted spacing between cysteine pairs. Despite this, spacing between the Cys(V) and Cys(VI) residues in the 7 LRs can vary up to two residues^28^. It is also remarkable that the Thr residue is only present adjacent to Cys(VI) in LR1^28^ which could suggest a special role for that amino acid in maintaining LR1 conformation, however, and as shown by our results, Thr62 replacement by a Met, does not impair LDLr activity. As shown by western blot and FACS analysis, p.(Thr62Met) LDLr variant is correctly expressed and retains a fully lipoprotein binding and uptake activity. This result can be expected because methionine side chains are relatively flexible, therefore allowing the local chain to adopt the correct LR1 structure. These results indicate that p.(Thr62Met) LDLr variant is a non-pathogenic LDLr variant.

Despite more than 2300 variants have been associated with FH (ClinVar database) and being one of the most common genetic disorders leading increased cardiovascular risk^2^, clinical diagnosis of FH is still understimated^7^. The small number of LDLr variants that have been functionally proved to cause FH so far is a good indicator of the importance of functional characterization because functional verification represents the best reference for providing a definite genetic FH diagnosis.

In conclusion, the data presented here puts into the spotlight the relevance of functional validation of LDLr variants to provide a definite clinical diagnosis of FH. We have assessed the activity of p.(Asp47Asn) and p.(Thr62Met) LDLr variants showing that neither of them is pathogenic. This is of special relevance because they are not the cause of the dyslipidemia found in their carriers, so further analysis should be done in these patients to find the real cause underlying FH. This *in vitro* functional validation that can be used routinely is necessary to identify the pathogenic variants that can enable more personalized treatments and improved prognosis.

## Electronic supplementary material

Suplementay Information
