# Supplementary material for: p.(Asp47Asn) and p.(Thr62Met): non deleterious LDL receptor missense variants functionally characterized in vitro
Source: Sci Rep. 2018 Nov 9;8:16614. doi: 10.1038/s41598-018-34715-x (PMC6226515; doi:10.1038/s41598-018-34715-x)
Supplement: Supplementary file 1 — Suplementay Information [file 41598_2018_34715_MOESM1_ESM.doc]

**SUPPLEMENTARY DATA**

Research article

**p.(Asp47Asn) and p.(Thr62Met): non deleterious LDL receptor missense variants functionally characterized *in vitro***

A. Benito-Vicente^1^, H. Siddiqi^1^, K.B. Uribe^1^, S. Jebari^1^, U. Galicia-Garcia^1^, A. Larrea-Sebal^1^, M. Stef^2^, H. Ostolaza^1^, L. Palacios^2^, C. Martin^1*^

**Author affiliations**

^1^Instituto Biofisika (UPV/EHU, CSIC) and Departamento de Bioquímica, Universidad del País Vasco, Apdo. 644, 48080 Bilbao, Spain.

^2^Progenika Biopharma, a Grifols Company, Derio, Spain.

*Corresponding Author E-mail: cesar.martin@ehu.eus. Tel. +34-94-601.80.53

**

**

**Supplementary Figure S1: Non processed Western blot signal. A) LDLr; B) GAPDH.** The figure shows all the bands, some bands do not appear because they are not related to this work. The image is not processed. The figure is divided in two panels because because after transference, the nitrocellulose membrane is cut to develop separately and simultaneously LDLr and GAPDH.
